# Comparisons of 7- to 78-joint ultrasonography scores: all different joint combinations show equal response to adalimumab treatment in patients with rheumatoid arthritis

**DOI:** 10.1186/ar3341

**Published:** 2011-05-27

**Authors:** Hilde Berner Hammer, Tore K Kvien

**Affiliations:** 1 Department of Rheumatology, Diakonhjemmet Hospital, Diakonveien 12, 0370 Oslo, Norway

## Abstract

**Introduction:**

The primary objectives were to explore the associations between a comprehensive ultrasonographic (US) assessment of joints, tendons and bursae and previously described reduced joint counts (7-, 12-, 28- and 44-joint score) as well as to assess the sensitivity to change of these different US joint combinations during biological treatment.

**Methods:**

Twenty patients with rheumatoid arthritis (RA) were examined by US (B-mode (BM) and power Doppler (PD)) with use of a semi-quantitative (0 to 3) score of 78 joints, 36 tendons/tendon groups and two bursae (hereafter described as the 78-joint score) at baseline and 1, 3, 6 and 12 months after initiating treatment with adalimumab. BM and PD scores for the different joint combinations were generated.

**Results:**

The reduced joint scores had high correlation coefficients with the 78-joint score at all examinations (range 0.79 to 0.99 for BM and 0.77 to 0.99 for PD, each *P *< 0.001) and sum BM and PD scores of all the different joint combinations improved significantly during follow-up (*P *≤ 0.05 to 0.001).

**Conclusions:**

The reduced joint combinations were highly associated to the 78-joint score. Furthermore, all the joint combinations presently explored responded well to biological treatment. This indicates that an approach focusing on few joints and tendons gives equivalent information about the inflammatory activity in RA patients as a comprehensive US examination. The optimal combination of joints and tendons for a valid, reliable and feasible US measurement should be further explored to define a US score for follow-up of RA patients on biological treatment.

## Introduction

Ultrasonography (US) is increasingly used for evaluation of synovitis in patients with inflammatory joint diseases, and synovitis and effusion are detected by grey scale (B-mode, BM) and vascularization by use of power Doppler (PD) [1]. US is a validated and reliable method for assessing joint inflammation [2-5] and it is sensitive to change during treatment with biological medication [5-8]. Different joint combinations have been proposed for optimal and/or feasible assessments of joint inflammation in patients with rheumatoid arthritis (RA). The 7-joint score by Backhaus *et al*. [9] was evaluated in a longitudinal multicenter German study and found to reflect disease activity and therapeutic response. Naredo *et al*. [10] developed from a 44-joint score in a multicenter Spanish study a 12-joint score that was useful in monitoring the response to biological medications. Another 44-joint score was used by Scirè *et al*. [11] and a 28-joint US score assessing the joints included in the composite DAS28 score [12] has been used in longitudinal and cross-sectional studies [3,5,13]. We have recently shown that US assessment of 78 joints also was sensitive to change during adalimumab treatment [14]. Thus, several joint combinations have been demonstrated to be useful in the assessment of RA patients, but further work is needed to identify the optimal combinations of joints for therapeutic assessment. In addition, tenosynovitis is a frequent pathology in RA patients and both US and MRI assessments have been shown to be responsive [15,16] and thus evaluation of tenosynovitis may be considered for inclusion in an optimal US score.

The objectives of the present longitudinal study of RA patients were to assess the associations between a comprehensive US score (including 78 joints, 36 tendons and 2 bursae) and existing reduced scores of 7-, 12-, 28- and 44-joints and to explore the responsiveness of the various joint scores during biological treatment.

## Materials and methods

A comprehensive US assessment of joints, tendons and bursae was performed in 20 patients with RA [17] (median (range) age 53 (21 to 78) years, disease duration 7.5 (1 to 26) years, 15 women, 70% IgM rheumatoid factor positive). US assessments of the 78 joints in these patients were previously described [14] as well as the wrist- and ankle tendon assessments [15]. In addition, the patients were examined bilaterally for tenosynovitis in the long biceps tendon and for inflammation in subdeltoid bursa. The patients were consecutively included the same day as they started treatment with adalimumab (40 mg every other week) as their first biological medication. All patients also received methotrexate and 14 patients used additionally prednisolone (median range dose: 7.5 (3.75 to 15) mg per day). US examinations were performed by one experienced sonographer (HBH) at baseline and after 1, 3, 6 and 12 months with use of a 5 to 13 MHz probe (Siemens Antares, Sonoline, Siemens Medical Solutions, 1230 Shorebird Way Mountain View, CA, USA) and fixed settings (PD with frequency 7.3 MHz and pulse repetitive frequency 391 Hz) [18]. The same machine and setting (without upgrading) was used throughout the study. The following joints were assessed bilaterally by use of standard longitudinal projections (scanning positions in parenthesis) [19]: proximal interphalangeal (PIP) 1 to 5 (dorsal), metacarpophalangeal (MCP) 1 to 5 (dorsal), carpometacarpal (CMC) 1 to 5 (CMC 1 radial/palmar, CMC 2 to 5 dorsal), wrist (each of the radiocarpal, intercarpal and radioulnar joints) (dorsal), elbow (anterior, lateral and posterior), shoulder (glenohumeral, with scanning dorsal transverse with maximal external rotation of the arm and acromioclavicular joints with longitudinal scanning), hip (anterior), knee (suprapatellar and lateral), ankle (talocrural joint) (anterior), four major foot joints (talonavicular, calcaneocuboidal, cuneonavicular, subtalar) (anterior and lateral), tarsometatarsal 1 to 5 (dorsal), metatarsophalangeal (MTP) 1 to 5 (dorsal) and the interphalangeal (dorsal) joint of the first toe (a total of 78 joints) [14]. In addition, tendons were examined bilaterally by use of definitions as described by OMERACT [1]. The extensor tendons of the wrists were assessed at the level of the radiocarpal joint and the examination included tendons of all six compartments: abductor pollicis longus and extensor pollicis brevis; extensor carpi radialis brevis and longus; extensor pollicis longus; extensor digitorum and extensor indicis; extensor digiti minimi; extensor carpi ulnaris. At the palmar side, three flexor tendons/groups were examined bilaterally at the level of the radiocarpal joint; flexor pollicis longus, flexor carpi radialis and combined flexor digitorum superficialis and profundus. In the ankles, eight tendons were assessed bilaterally: peroneus longus and brevis (behind the lateral malleol), extensor digitorum longus, extensor hallucis longus and tibialis anterior (anterior at the level of the distal tibia), tibialis posterior, flexor digitorum longus and flexor hallucis longus (behind the medial malleol) [15]. The long biceps tendon (anterior) was also examined, and thus a total of 36 tendons/tendon groups were evaluated. All tendons were assessed transversely by moving the probe proximally and then distally along the tendons and finally longitudinally by moving the probe medially and laterally over the tendon/tendon groups. In addition, potential abnormalities in subdeltoid bursa were explored bilaterally by use of a transverse scan at the level of tuberculum major, and the size and PD activity were evaluated using additionally scans, as required. The joints, tendons and bursae were scored for BM (presence of synovitis and joint fluid) and PD (presence of vascularization) as score 0 = none, score 1 = minor, score 2 = moderate or score 3 = major presence.

All US examinations were performed in one room in the morning, after at least half an hour of acclimatization to room temperature [20]. The pressure of the probe was as low as possible to obtain optimal PD signals. The hands and fingers were assessed while resting on a small table, the elbows were examined in extension and flexion, the shoulders with the patient sitting and the lower limbs were examined with the patient lying on a bench. The US examiner was blinded for previous US results as well as for the results from clinical and laboratory examinations the same day.

Reliability tests for US scoring of joints were performed on acquired images with median (range) intraobserver intraclass correlation coefficients (95% CI) of 0.97 (0.96 to 0.98) for BM scores and 0.98 (0.97 to 0.99) for PD scores [14]. No reliability tests were, however, performed for the scoring of tendons and bursae.

With the BM or PD scores from the comprehensive assessment of 78 joints, 36 tendons or tendon groups and 2 bursae (hereafter called the 78-joint score) as basis, sum scores of BM or PD for the different joint combinations/joint scores were computed. The 7-joint score by Backhaus *et al*. includes examination of the wrist, MCP 2 and 3, PIP 2 and 3, and MTP 2 and 5 at the clinically dominant side with assessment of synovitis, paratenonitis/tenosynovitis and erosions [9]. However, neither examination of flexor tenosynovitis in the second and third finger nor assessments of erosions were performed in this study, and joints and tendons on the patients right side were presently defined as being the dominant side and included in the calculations of the 7-joint score. The original 7-joint score includes assessment of BM synovitis in MCP 2 and 3 only from the palmar view, paratenonitis/tenosynovitis from both dorsal and palmar aspect, and PD assessments on both palmar and dorsal aspect of these joints. The PIP two and three joints were in the 7-joint score assessed for synovitis only on the palmar aspect while PD assessments were performed both at dorsal and palmar side. In our study only dorsal scans were performed on MCP and PIP joints and thus these scores were included in the present calculations. The wrist was in the 78-joint score examined as the radiocarpal, intercarpal and radioulnar joints separately, and the sum of these scores was included as the wrist-assessment in the 7-joint score. The tendons described as dorsal and ulnar part of the wrist in the 7-joint score were in this study defined as compartment 4 and 6 of the extensor tendons, and the palmar tendons were defined as the superficial and deep flexor tendons, and they were all included in the sum 7-joint score.

The 12-joint score by Naredo *et al*. [10] includes bilateral examination of the elbow, wrist, MCP 2 and 3, knee and ankle in addition to the medial and lateral tendon compartments in the ankle. In the original 12-joint score the wrist was described as the dorsal carpal recess, which was presently defined as the radiocarpal and intercarpal joints. Except for both palmar and dorsal evaluation of MCP 2 and 3 joints in the 12-joint score, the scannings of joints and tendons were similar to the 78-joint score.

The 28-joint score [5,13] includes the same 28 joints as used in the DAS28 score [12] with bilateral examination of the glenohumeral joint, elbow, wrist, MCP 1 to 5, PIP 1 to 5 and knee with similar scanning method as used in the 78-joint score. However, since the wrist score was not clearly defined, the highest score of the radiocarpal, intercarpal and radioulnar joints was used in this study for the calculation of sum scores for BM and PD of these 28 joints.

The 44-joint score by Naredo *et al*. [10] includes the glenohumeral joint, elbow, wrist, MCP 1 to 5, PIP 1 to 5, hip, knee, ankle, midtarsal joints and MTP 1 to 5 joints. From the 78-joint score we used the highest score of the three wrist joints and the highest score of the midtarsal joints for the calculations of the 44-joint score. In addition, the 44-joint score includes the biceps, dorsal and palmar wrist tendons, medial, anterior and lateral ankle tendons as well as the subdeltoid bursae. Thus all these tendons and bursae were included in the present calculations. Table 1 displays an overview of the joints, tendons and bursae included in the different reduced joint scores.

**Table 1:** The joints, tendons and bursae included in the 7-, 12-, 28- and 44-joint scores.

Reduced joint score	Joints, tendons and bursae included
7-joint score [9]	On the clinically dominant side: Wrist, MCP 2 and 3, PIP 2 and 3, MTP 2 and 5 Dorsal, ulnar and palmar tendon sheaths of the wrist, flexor tendon sheaths of 2 and 3 finger, paratendonitis dorsal 2 and 3 finger
12-joint score [10]	Bilateral examination of: Elbow, wrist, MCP 2 and 3, knee and ankle Medial and lateral tendon sheaths of the ankle
28-joint score [12]	Bilateral examination of: Shoulder (glenohumeral), elbow, wrist, MCP 1 to 5, PIP 1 to 5 and knee
44-joint score [10]	Bilateral examination of: Shoulder (glenohumeral), elbow, wrist, MCP 1 to 5, PIP 1 to 5, hip, knee, ankle, mid-tarsal, MTP 1 to 5 Biceps tendon sheath, extensor and flexor tendons sheaths of the wrist, flexor tendons sheaths of the fingers, anterior, medial and lateral tendon sheaths of the ankle Subdeltoid bursa

MCP, metacarpo-phalangeal joint; PIP, proximal inter-phalangeal joint; MTP, metatarso-phalangeal joint

The patients gave written consent according to the Declaration of Helsinki, and the study was approved by the local ethics committee (the regional committee for medical and health research ethics (REK), South-East).

### Statistics

Wilcoxon signed rank test was used to examine changes in US sum scores during follow-up for the different joint combinations. Associations between the sum scores of BM or PD of the comprehensive joint score and the different reduced joint combinations (described in Table 1) were assessed by use of Spearman's rank correlations. A *P*-value < 0.05 was considered statistically significant. Responsiveness was visualized by use of simple error bar plots with 95% confidence interval.

## Results

PIP3, MCP 1 and 2, CMC1, radiocarpal- and radioulnar joints, MTP 1, 2, 3 and 5 were inflamed (BM ≥ 1) at one or both sides in ≥ 50% of the patients at baseline. High BM scores (2 or 3) were found in MCP 1 and 2, CMC1, radiocarpal- and radioulnar joints, MTP 1, 2 and 3 at one or both sides in ≥ 40% of the patients and high PD activity (score 2 or 3) was detected in MCP 1, 2 and 5, CMC1, radiocarpal- and radioulnar joints, MTP 2 and 5 at one or both sides in at least 30% of the patients. Thus, all the different joint combinations include most of the joints which were found to be frequently inflamed.

High correlations were found between the sum BM 78-joint score and all of the different joint combinations, with median (range) correlation coefficients at the five examinations of 0.89 (0.86 to 0.96) for BM 7-joint score, 0.86 (0.79 to 0.96) for BM 12-joint score, 0.95 (0.92 to 0.97) for BM 28-joint score and 0.97 (0.95 to 0.99) for BM 44-joint score (each *P *< 0.001). High correlations were also found between the sum PD 78-joint score and all of the joint combinations with median (range) 0.85 (0.80 to 0.95) for PD 7-joint score, 0.81 (0.77 to 0.90) for PD 12-joint score, 0.93 (0.89 to 0.95) for PD 28-joint score and 0.98 (0.95 to 0.99) for PD 44-joint score (each *P *< 0.001). Tables 2 and 3 show the correlation coefficients between all the joint combinations at baseline and at the 12-month follow-up.

**Table 2:** Correlation coefficients between sum scores B-mode (BM) of the different joint combinations

	BM 12-joint score	BM 28-joint score	BM 44-joint score	BM 78-joint score
BM 7-joint score	0.87*/**0.92***	0.86*/**0.89***	0.85*/**0.91***	0.87*/**0.93***
BM 12-joint score		0.84*/**0.93***	0.86*/**0.96***	0.87*/**0.96***
BM 28-joint score			0.94*/**0.93***	0.95*/**0.93***
BM 44-joint score				0.97*/**0.99***

Spearman's rank correlation coefficients between sum scores BM of the different joint combinations at baseline and at 12 months follow-up (in bold italics) (*P *< 0.001 for all the correlations).

**Table 3:** Correlation coefficients between sum scores power Doppler (PD) of the different joint combinations

	PD 12-joint score	PD 28-joint score	PD 44-joint score	PD 78-joint score
PD 7-joint score	0.89*/**0.89***	0.92*/**0.94***	0.94*/**0.93***	0.95*/**0.95***
PD 12-joint score		0.81*/**0.85***	0.86*/**0.85***	0.86*/**0.89***
PD 28-joint score			0.94*/**0.95***	0.93*/**0.95***
PD 44-joint score				0.95*/**0.98***

Spearman's rank correlation coefficients between sum scores PD of the different joint combinations at baseline and at 12 months follow-up (in bold italics) (*P *< 0.001 for all the correlations).

**Figure 1:**
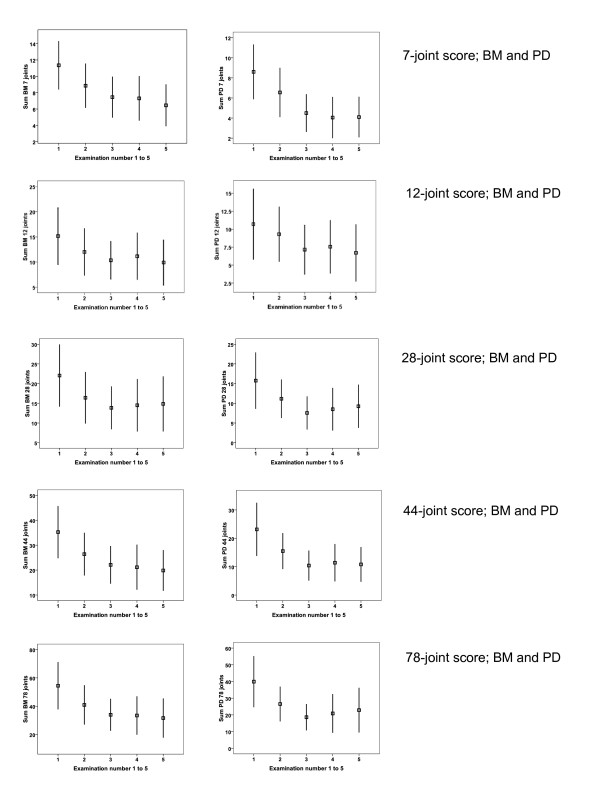
**Error bar chart of the 7-, 12-, 28-, 44- and 78 joint scores for the mean sum BM or PD scores (with 95% confidence interval)**. BM; B-mode, PD; power Doppler

All the joint combinations showed significant improvement in sum scores of BM and PD during treatment (Tables 4 and 5), which is illustrated by use of error bar plots of the sum BM or PD scores at all examinations (Figure 1). In particular, the more comprehensive joint counts did not seem to have superior responsiveness compared to the counts with inclusion of fewer joints/tendons.

**Table 4:** Sum B-mode (BM) scores of the different joint combinations during the study

	Baseline	1 month	3 months	6 monhts	12 months
**7-joint score**	11.5 (1 to 22)	7.5 (0 to 20)*	6.5 (0 to 15)*	7.0 (0 to 17)**	5.5 (0 to 16)**
**12-joint score**	15.0 (0 to 52)	12.5 (0 to 37)*	11.0 (0 to 28)*	9.5 (0 to 32)*	7.5 (0 to 28)*
**28-joint score**	17.0 (2 to 65)	13.0 (2 to 57)**	11.0 (0 to 45)**	8.5 (1 to 45)*	12.0 (0 to 45)*
**44-joint score**	40.0 (6 to 131)	31.5 (3 to 103)**	29.5 (6 to 71)**	19.5 (1 to 69)**	20.0 (1 to 73)**
**78-joint score**	53.5 (6 to 154)	38.5 (3 to 124)**	36.5 (6 to 96)**	23.5 (1 to 91)**	27.0 (1 to 101)**

Sum BM scores of the different joint combinations at baseline and after 1, 3, 6 and 12 months. All joint combinations showed significant change from baseline (* = *P *≤ 0.05, ** = *P *≤ 0.001).

**Table 5:** Sum power Doppler (PD) scores of the different joint combinations during the study

	Baseline	1 month	3 months	6 monhts	12 months
**7-joint score**	8.0 (0 to 19)	5.0 (0 to 17)*	3.5 (0 to 13)*	2.0 (0 to 12)**	2.5 (0 to 12)**
**12-joint score**	9.0 (0 to 42)	8.0 (0 to 31)	6.5 (0 to 26)**	6.0 (0 to 29)*	3.0 (0 to 29)*
**28-joint score**	10.5 (0 to 50)	8.0 (0 to 34)*	3.5 (0 to 28)**	3.5 (0 to 35)**	4.0 (0 to 35)*
**44-joint score**	26.5 (1 to 96)	17.0 (0 to 63)**	11.5 (1 to 38)**	12.0 (0 to 63)**	9.5 (0 to 73)**
**78-joint score**	33.0 (1 to 119)	21.0 (0 to 83)**	15.5 (2 to 52)**	13.5 (0 to 82)**	12.5 (0 to 81)**

Sum PD scores of the different joint combinations at baseline and after 1, 3, 6 and 12 months. All joint combinations showed significant change from baseline (* = *P *≤ 0.05, ** = *P *≤ 0.001).

## Discussion

To our knowledge the present longitudinal study with examinations of a large number of joints, tendons and bursae is the most comprehensive US assessment published so far. During biological treatment the US scores based on a reduced number of joints and tendons were found to have high correlations with the comprehensive score at all examinations and to provide comparable information to the comprehensive score regarding responsiveness. In addition, we found that the most frequently inflamed joints with high degrees of BM and PD pathology were included in the described reduced joint counts. The CMC 1 joint, however, is often involved in osteoarthritis [21,22] and this joint may not be useful for evaluation of RA activity. This joint is also not considered in the new ACR/EULAR classification criteria for RA [23].

The present US assessments of joints and tendons were slightly different from some of the reduced joint scores. The most important difference was the scanning method of MCP and PIP joints, where the 7-joint score assesses both the palmar and dorsal side, while only dorsal assessments were performed in the 78-joint score. There is so far no consensus on which side of these finger joints US examinations give the most sensitive and/or reliable results, but up to now the majority of studies have assessed the dorsal part of the finger joints.

The PD scores in the 78-joint examination were defined as none, minor, moderate or major presence of PD activity. The PD scoring in the reduced joint scores were slightly different and performed as described by Szkudlarek *et al*. [24,25] who define score 2 or 3 as a PD signal covering less or more than 50% of the synovitis area. However, it may be difficult to differentiate between these scores since most machines do not have programs defining a certain percentage of PD activity relative to grey scale pathology.

The various scores that are explored in this study have been evaluated in longitudinal studies and are found to detect improvement during biological medication. So far, most evaluations of response to treatment have been performed by use of the patient's evaluations, clinical examinations and laboratory assessments, and with presentation of composite scores that reflect inflammatory activity [12,26-28], in accordance with the published EULAR/ACR recommendations [29]. US has in previous studies been found to be a valid and reliable examination for assessment of synovitis as well as the degree of vascularization (by use of PD activity) [2-5]. Since the primary goal for RA management is to suppress the inflammation to a level that prevents disability and joint destruction [30], US may be an additional promising clinical tool for evaluation of response to treatment and level of inflammatory activity. However, to make US a feasible method in the clinical setting, it is of major importance to explore the lowest number of joints and tendons that is able to give information about the inflammatory process in most of the patients. The present study indicates that assessment of as few as seven joints and five tendons/tendon compartments is sensitive to change and with high correlations to the comprehensive joint and tendon assessment. The inclusion of a few tendons is supported by a recent study where the extensor carpi ulnaris and tibialis posterior tendons were found to be most frequently inflamed and with US scoring of these tendons being highly sensitive to improvement during treatment [15]. Thus further studies ought to be performed to identify the joints and tendons that should be included in a limited US score.

In a study of RA patients in clinical as well as ACR and DAS28 remission, the majority of patients had US evidence of inflammation when eight joints were assessed (dominant hand MCP 2 to 5 and four wrist joints) [31]. Thus, this low number of joints was able to detect ongoing pathology in spite of normal combined clinical and laboratory composite scores. In the search for an optimal combination of joints and tendons for US scoring of RA patients, this scoring system should also be explored for its sensitivity to evaluate US remission in RA patients.

The strength of the present study is the performance of a comprehensive joint, tendon and bursae assessment by only one experienced sonographer in a longitudinal design during anti-TNF treatment, use of standardized US assessments utilizing the same machine throughout the study and no missing examinations. However, an obvious weakness is the low number of participating patients. For this reason we were not able to use the dataset to identify a new US joint combination with high trade-off between feasibility and responsiveness. Nevertheless, in spite of the low number of patients included, the present study was able to detect significant associations between the different joint scores as well as significant improvements in all the US scores.

## Conclusions

The joints found to be most frequently inflamed in the present study are to a large extent included in the different described reduced joint combinations. All the reduced joint scores were found to have high associations to the comprehensive 78-joint score and they were sensitive to change during biological medication. Even the 7-joint score was as sensitive to change as the 78-joint score for BM and PD assessments. The optimal combination of joints and tendons to be assessed by US should be further explored to make a valid, reliable and feasible score. However, the present study supports that even low numbers of joints and tendons seem to be sufficient to reflect the response to biological treatment.

## Abbreviations

ACR: American Congress of Rheumatology; Anti-TNF: anti-tumor necrosis factor; BM: B-mode; CI: confidence interval; CMC: carp-metacarpal; DAS28: disease activity score 28 joints; EULAR: European League Against Rheumatism; IgM: immunoglobulin M; MCP: metacarpo-phalangeal; MHz: megahertz; MRI: Magnetic Resonance Imaging; MTP: metatarso-phalangeal; OMERACT: Outcome Measures in RA Clinical Trials; PD: power Doppler; PIP: proximal interphalangeal; RA: rheumatoid arthritis; US: ultrasonography

## Competing interests

The authors declare that they have no competing interests.

## Authors' contributions

HBH has been in charge of the conception and design, has performed all the US assessments and the analysis of the data, has been in charge of drafting the manuscript and has given final approval of the manuscript version to be published. TKK has made substantial contributions to the design of the study and interpretation of data, has revised it critically for important intellectual content and has given final approval of the version to be published.
